# The Hospital Nursing Supplement

**Published:** 1893-09-23

**Authors:** 


### "The Hospital" Nursing Mirror.

BEING THE EXTRA NURSING SUPPLEMENT OF "THE HOSPITAL" NEWSPAPER.

[Contributions for this Supplement should be addressed to the Editor, THE HOSPITAL, 428, Strand, London, W.C., and should have the word "Nursing" plainly written in left-hand top corner of the envelope.]

### News from the Nursing World.

### OUR EXAMINATIONS.

WITH the end of the holidays we have resumed our examination questions, and we propose to continue to pay special attention to such practical points as come, sometime or other, within the experience of all of us. We do not limit ourselves to any particular line of nursing, our aim being to encourage all attendants on the sick, to think for themselves, and to know what to do in every emergency, whether great or small. To carry out the doctor's orders in hospital wards requires merely intelligent obedience, but to do the best possible thing for rich or poor sick people in their own homes is vastly more difficult. Therefore, our readers are asked to understand plainly that we want their answers to show us exactly what each correspondent would do if she had to act on her own responsibility, either in the absence of or pending the arrival of the doctor, to whom of course the responsibility as well as the treatment of cases of illness always belongs. Sometimes a correspondent demands from us a definition of how, when, and where preliminary knowledge should be obtained? Our examinations are just the same as others, and therefore intending competitors, having learnt all they can, practically and theoretically on each subject, have then only to reduce their knowledge into an accurate and concise written form.

### TEACHER AND TAUGHT.

"WHAT do I teach, or how much do they learn, which is it you wish to know?" was a reply which caused its recipient to rejoin very thoughtfully, "Both, if you please." Consequently the experienced lecturer proceeded to explain that he fancied he knew pretty accurately what he said to his classes, but until the questions, always encouraged at the end of each address had been asked, he was quite ignorant of the impressions conveyed to unprofessional minds by professional teaching. For example, some ladies had recently asked to be told what they ought to do if a person cut his throat. An explanation was thereupon given to the whole class, of the requisite posture, &c., to be assumed. All were interested, and seemed intelligently impressed, and also by the medical lecturer's subsequent simple directions as to temporary arrest of hæmorrhage. A few days later a man injured himself horribly with a scythe in a cornfield. The wound inflicted was on the back of the neck, and there were several persons within reach who had been present at the lecture, but not a single one dared touch the unhappy man until the doctor's arrival, because they said the wound was at the back, not in front, as they had been taught to expect. "Was teaching or learning to blame for this?" grimly queried the lecturer.

### ELASTIC LIMITS.

"ALLOW me to say a few words," or "I wish to say a few words," are conventional introductions to addresses lasting twenty minutes and to letters which cover several sheets of foolscap. Speeches victimise audiences, but MS. appearing in letter form becomes an editorial matter. If professional and unprofessional correspondents would content themselves with literally "a few words," containing the pith of the matter they wish to discuss, space could often be spared, and a great variety of opinions secured. Instead of this, numbers of voluminous so-called "letters" flood the post, and contributors are found bewailing the ill-luck which attends their epistolary efforts. Surely it is a little unreasonable to expect ten or sixteen sheets of manuscript to gain insertion in any journal which is not lamentably in want of free "copy." Concise and interesting letters to an editor will always command attention, but for the bulky sheets which appear under that inappropriate title the inevitable destination must be the waste paper basket.

### CRITICAL OBSERVERS.

"WHAT to observe" is one of the first instructions given to a probationer in connection with hospital patients. Unless she is taught this promptly she will waste time in observing immaterial and overlooking many important facts. Experience alone renders observation instinctively accurate, and this applies as much to institutions as to persons. The ordinary visitor notices the colouring of the walls, the books supplied to the patients, the appearance and manners of the nurses, and mayhap the quality of the food supply. A "professional" visitor, whether matron or ward nurse, "going round" any hospital observes a great deal more than this. She notes not only the food, but the way in which it is given to the patient—whether his knife, fork, and bread are placed by him before his hot dinner, or if inattention to details leaves these to follow haphazard in the wake of the plate of meat and vegetables, which grows cold and greasy meanwhile. The professional visitor cares more for the bath-rooms than for the books, although not contemptuous of the latter. She knows well that the position and condition of baths, lavatories, &c., are a sure criterion of the nursing work carried on in a ward.

### WELL CARED FOR.

THE pleasant house opened this year under the title of St. Catherine's Home at Bradford is proving worthy of its name. It is, indeed, a haven of refuge to those afflicted with tedious and incurable diseases. Their comfort is studied in every detail, life being made as endurable and death as easy as can be accomplished by medical skill and trained nursing. Kindliness and consideration appear to be the watchwords of the committee and the matron. An extra nurse is secured for short periods, when the critical condition of a patient necessitates especial attention. The engagement of an additional nurse whenever required should be an invariable rule in all small hospitals. We hope the number of establishments is limited where there prevails such a system as that at Watford District Cottage Hospital,, of which the late matron wrote last week that she "could not leave critical cases to untrained care, and was therefore sometimes obliged to nurse a patient day and night for a week together, without any proper rest."

### WORTHY OF IMITATION.

Walls and ceilings of white tiles, floors of red tiles, and baths of white porcelain form an attractive picture, and nobody is surprised to find that visitors ejaculate, "Lovely, indeed!" when they see this pretty combination at the Cork Hospital for Women and Children. The new operating theatre is to be lined with white tiles, and will possess every modern improvement. The Matron, Miss Baxter's "special collection" has furnished the funds for this new bath-room wing. She may well also be proud of the nurses' dining-room, with its polished floor and rich-hued oriental rugs. Small tables dotted about accommodate four or six nurses at each, and the vases of fresh flowers are as attractive as the white and shining condition of the linen, the plate, and the crockery.

### A FRENCH DOCTOR'S WAITING-ROOM.

The doctor can be consulted from one to three, the solemn manservant assures the stranger, as he invites "Monsieur" to enter. Instead of the heavily furnished dining-room with its large table and rows of chairs so familiar to English patients, a smartly-furnished drawing-room receives the Parisian consultant's clients. Each piece of highly-polished or inlaid furniture is entirely covered with ornaments or curiosities, until it seems as if there were no space to add one more to their number. There is a heap of papers in one place and several piles of books on a table, but daily papers are not spread abroad as in England, where patients have the opportunity of burying their anxieties in a nervous perusal of *Punch*, the *Illustrated London News*, or the *Idler*. In Paris people converse together or else silently observe their companions until the inner door opens and the first arrival is requested to enter. The chair which awaits the patient in the Englishman's consulting-room is so plainly intended for him that he lacks courage to take a less conspicuous position, and he sinks down at once, feeling as if he were in a photographer's studio, confronted however, by a far keener gaze than that of any artist. In Paris, whilst several chairs seem to invite the visitor to be seated, neither of them appears to possess any special indication for selection.

### INDIA.

There is a great deal of remittent fever at Umballa, and some very bad cases of enteric fever, and the nursing sisters are kept fully employed. They are also liable at any time to be sent off to meet emergencies at other places where sisters are not permanently stationed. Therefore, nursing in India is sometimes made up of a great variety of experiences.

### AUSTRALIA.

A concert was given recently at Melbourne, in the Exhibition Buildings, in aid of the united hospitals, and seven thousand people were present. A large number of persons were unable to obtain admission. The arrangements were admirably carried out by the Victorian Police Force Band, the 18th Regiment and the Victorian Rangers. The receipts amounted to over £300, which will be handed over to the hospitals.

### OUR AMERICAN SISTERS.

The origin and work of the Queen Victoria's Jubilee Institute for Nurses is admirably set forth in the paper which was read by Miss A. Hughes at the Nursing Congress at Chicago, and which appears in this month's issue of the *Trained Nurse*. As Superintendent of the Metropolitan and National Nursing Association, Miss A. Hughes is able to speak with authority of the training in district work which the Queen's nurses receive, and all interested in the plans and organistion of the institute will take as much pleasure in reading, as the audience at Chicago exhibited in listening to the paper in question. The same number of the magazine gives an article by Miss C. E. M. Somerville on "District Nursing in America," which shows in its record of one day's work that human nature is much the same in both hemispheres, and a woman with a scalded face is described as applying ink for a primary dressing by advice of a neighbour. Without doubt other harmful substances are ignorantly made use of there as here. That the eaching of domestic sanitation is quietly making good progress in Boston is clearly demonstrated by the District nurse's satisfactory statement that "almost every day I find some former patient carrying out many of the simple directions that have been given during some former sickness."

### SHORT ITEMS.

The new Hospital for Women at Nottingham will be shortly transferred to the commodious buildings which have been purchased for and adapted to the purpose. Lady Belper has consented to perform the opening ceremony on October 12th.—The Cottage Hospital at Mull, N.B., has already proved so valuable to the scattered population on both sides of the Sound, that it is under discussion to further benefit the sick poor by the establishment of a district nursing association, which might eventually be affiliated with the Q. V. J. Institute.—People willing to help hospital funds should be very careful to see that they place their donations in reliable hands. The collecting boxes which are aggressively flourished and shaken before the unwary passenger are by no means invariably to be trusted. It is the easiest thing possible to "get up" a box effectively, and the temptation to do so has led to frauds. Therefore, whether the gift bestowed be a pound or a penny, it should be placed in the official boxes permanently established within or without the hospital walls, or else be delivered direct to the Secretary.—The two hospital sisters who went out to Labrador in connection with the Mission to Deep Sea Fishermen arrived safely, and are very busy amidst their novel surroundings. It is uncertain whether the hospital will be ready for occupation before the new year.—Mr. Knight, of Farnham, Surrey, has presented the land for the cottage hospital which is shortly to be erected with the money (£1,500) left for the purpose by the late Mrs. George Trimmer, and which will be called the "Trimmer Cottage Hospital."—An article by Princess Christian on the Royal British Nurses' Association is advertised to appear in *Atalanta* of October.

### Trained Nursing in Berlin.

*Prepared for the International Congress of Nurses.*

### THE VICTORIA HOUSE.

WHEREAS the Victoria House has its own training school in which the nurses can receive their technical training, it entered in 1884 into an agreement with the authorities of the city of Berlin by which the Victoria sisters could receive their practical training at the city hospital of Fredrichsheim. By this arrangement the Victoria House promised two-thirds of their trained nurses for different city hospitals. In this way those who have received their first six months' training are eligible to serve in the city hospitals. The Victoria House receives a yearly income of 450 marks from the city of Berlin for each head nurse in the city hospitals, and 360 marks for each sister, and these sums of money are divided among the sisters according to the agreement of the authorities of the Victoria House. Besides the 100 sisters who are now serving in the city hospitals, the Victoria House has a number of State and private institutions and associations to supply, partly to take charge of the sick, and partly to direct and superintend. In the former case they have male and female attendants who assist, but do not belong to the Victoria House. The compensation which is received at the Victoria House from these institutions amounts approximately to 500 marks yearly for each nurse, whether it be in the capacity of nurse or of superintendent. No difference is made in the salaries of the Victoria sisters, whether they work as nurses or superintendents, for each one is expected to be placed in the position she is best fitted for, and as such to do their work to the best of their ability. The income of the Victoria House up to 1893 consists of the earnings of the sisters, which amounted to 57,471 marks, besides presents and dues of members up to the present time, and the interest on 120,000 marks. This sum was a present from the Crown Prince and Princess of the German Empire to the Victoria House; this amount was presented to them in 1883 by the city of Berlin on the occasion of their silver wedding; they gave it for the building of a house for the Victoria sisters. It was not begun until 1892. The constant increase of the associations made the erection of a new building at last absolutely necessary. The city of Berlin appropriated 130,000 marks to the above sum and a building site, with the condition that the house should remain city property. The present home for the nurses is near the city hospital at Friedrichsheim, in which the superintendent and pupil nurses have resided since 1884; they had also rented quarters in this neighbourhood for private and sick nurses. The new Victoria House, will be located further out in the suburbs, near the hospital at Friedrichsheim. From October 1st twenty to thirty sisters will live here during the first six months of their training.

There are thirty-six rooms in this building for private and sick nurses, besides dining, sitting, reading, and meeting rooms for all the sisters of the Victoria House. There they meet at least twice a year at the anniversary of the opening and at Christmas, and here they find a kind home and every care in time of sickness and in the need of rest. All the Victoria sisters who are engaged in hospital work live and board there, and also the sixty-one sisters who are at present employed at the Friedrichsheim Hospital who have hitherto lived at the hospital itself.

For each nurse the city of Berlin pays one mark per day, the same sum which it used to cost to board them. The sisters are responsible to the doctors in chief, and to the assistant doctors in all their work in connection with the different institutions and associations. At the head of each district there is a head nurse, who is assisted by subordinate sisters and pupil nurses, for whom she is responsible for the conscientious fulfilment of duties. In the larger hospitals one of the head nurses acts as assistant to the superintendent; she is free in this capacity to give afternoons off duty to the sisters and pupil nurses, and her advice is solicited in all difficulties. In circumstances too difficult for her to decide, she applies to the superintendent for her decision; in any case, she is required to consult the superintendent in all matters regarding the sisters of the institution. The duties of the nurses are equally arranged; the work is divided into day and night duty. The hours of duty in most of the hospitals is from six a.m. to eight p.m. The night duty from eight p.m. to nine a.m. If the night duty finish their work they may go off duty earlier. As a rule the night staff is changed every month, and for the sake of the nurses' health they are not required to go on night duty more than four times a year. Before going on duty at night they have supper all together. During the night they have a similar meal, which they take in the kitchen of the ward. They take a cup of coffee with the day nurses, and at ten o'clock they have breakfast when they come off duty. From that time until twelve o'clock they generally spend out in the grounds of the hospital. They then sleep for seven hours. Twice a week they are allowed to go out from nine until one p.m. The day nurses have one hour off duty daily, besides nine hours free weekly. The pupil nurses are required to be in the Victoria House by ten p.m. Whatever comes under the head of nursing is in the hands of the nurses, but all other work in the way of house cleaning is done by men and women employed for that purpose. Orderlies are given the care of from 20 to 40 male patients; they assist in keeping them in good order, in lifting and in carrying. This assistance is provided for the sisters by the different institutions, and not by the Victoria House. In regard to the uniform, they all wear the same on duty. It consists of a light blue wash dress, white apron, and white cap. On Sundays they wear a dark blue cashmere dress. In the street they wear a plain black mantle made in the latest style, and a small black inconspicuous bonnet, faced with white ruching and white ties. When the nurses are off duty they can wear what they please. They are allowed to attend concerts and theatres, but cannot go to public places alone, and must inform the superintendent or assistant of their intention of going.

There are no other restrictions, and it is expected that young women of their standing should conduct themselves as ladies. If they ever take advantage of these liberties they are expelled from the society. In the Victoria House no one is obliged to attend religious services. On Sunday everyone has an opportunity of attending the Protestant service held in the building. They are allowed to attend services outside the hospital, provided this does not interfere with their duties, for it is considered that services to the sick is service to God. The majority of the sisters are Protestants. At present there are 171 sisters, out of which number 158 are Protestants and Roman Catholics, and four belonging to other denominations. So far there has never been any religious discord in the Victoria House.

### Wants and Workers.

*X. Y. Z.* will be glad to hear of any asylums for the insane, other than pauper ones, where ladies in destitute circumstances are admitted without payment.

WILL anyone kindly tell a nurse, who is suffering from eczema on the hands, of a home where she could receive a little attention until her health is restored?—*Nurse Emily.*

A YOUNG girl, now aged 15, has never had any growth of hair, consequently has to obtain a wig. Can anyone give information of any society that will help her parents in procuring one, as the expense is beyond their means?—*F. R. S.*

### The Parish Nurse.

### II.—HER POSITION AND DUTIES.

In a paper which appeared in The Hospital some weeks ago I gave a short general sketch of the daily life and duties of a parish nurse. Having had some queries put to me since, I should like to add a few words as to points which I did not then touch upon. How such a work may be begun? And if by subscriptions or payments demanded, as in an ordinary nursing home?

In the first place, I should not advise any nurse to set up for herself and take subscriptions—still less money from the poor. This plan might be, and I believe has been, carried out in maternity work, because there you have a previously-established practice of payment for attendance during a specified time. But in cases of ordinary sickness it would be very difficult, if not impossible, to work such a system.

Appointments are usually made by the vicar of a parish or a committee of ladies with authority from him, the committee sometimes being appointed by a meeting of the townspeople. The advantages of despotism *versus* constitutional government, is a matter each nurse must decide according to her tastes and politics. On the one hand, she may suffer from too much supervision; on the other, she may feel the want of help. She is responsible to many in the one case, but she has usually, if possessed of some amount of tact, the help and kind co-operation of many. In the other case, the vicar's nurse has but one person to please or displease; and by his sole *fiat* she stands or falls. The salary runs from £50 to £70, with or without rooms. It is not magnificent, and it is not equal to the hospital rate, with board, lodging, and uniform provided. But we may suppose the nurse prefers the greater freedom and less exhausting work, and is prepared to make a monetary sacrifice to obtain occupation which is useful and more suited to her powers.

With untrained nurses we have really nothing whatever to do, but a word may be said as to the "District Nurses" sent out by an excellent religious society, the Church Army. They are good women, and most useful in their own line, but they are not in any real sense of the word trained nurses. Yet they are, if not sent out, certainly accepted as such by outsiders. For three months only, they are non-resident helpers in a hospital; they are then considered to be fully instructed, and go out to country districts. One of them, a most capable woman, once deplored to me this painful want of knowledge and experience. All honour to her that she shortly after threw up her appointment, and went back to hospital for further training. The fact speaks for itself.

Below, and far below these again, are the Bible-woman and the salaried district visitor, who both set up to do a little nursing. One can hardly blame them, poor things! that they will do anything for a livelihood. They will tell you they have had "a deal of sickness in their own families"—*ergo*, they are qualified nurses. As well may the "clever young man" at at the chemist's shop diagnose a case, and "make up a bottle for it."

But granted the usual three years' training, still more experience even than this, is at least advisable. It shows an utter ignorance of the peculiar circumstances of the work to argue that because there is not always a great deal to do, and the cases are not exceptional, or often surgical—for such are usually sent away for treatment—therefore any tyro will suffice. It is the isolation, and the absolute necessity for self-dependence and self-reliance, which make it obligatory to have a practised hand. Of course, the nurse is working under a doctor—if she does not do so, she splits upon a rock at once, but of this hereafter—but where is the doctor? At his home three miles away, or possibly out on his round six miles in the opposite direction. Everything depends on the nurse. She cannot run and call the Ward Sister, nor invoke the aid of the House Surgeon. She must decide herself, directly she sees her patient. Also, she has to consider what can best be done—with the often most unhopeful materials before her. If she has never chanced to see a case of this kind before, how can she form an opinion as to the state of her patient—how much of the suffering is normal and inevitable, how much is preventable; probably everything she wants is conspicuous by its absence. What she has not with her in her bag she must send home for—linseed or what not. If she has no bump of management, after waiting half an hour helplessly, she will receive the discouraging reply that there is no more in the medicine cupboard, and there will be another delay. So much depends upon her forethought, patience, ingenuity, and—last but not least—actual previous contact with the very poor in their own homes. A nurse may be a shrewd and clever woman, a lady at heart as well as in name, overflowing with love and kindness, and yet she may be a hopeless failure. She has spent some time in a London hospital, receiving daily patients of the poorest class; she has had, perhaps, experience in a workhouse infirmary; and still these tell her nothing of the inside of a cottage home. Her sole acquaintance with this is an occasional cursory glance round the best parlour. In a hospital, however rude and rough the patients may be naturally, they are cowed by their unusual surroundings. They are thankful for any relief to their pain, and they are compulsorily clean. If you consider a measure advisable for their well-being, it is ordered and done. In the parish there are the relations to consult, three or four, all with a different opinion on the subject, and preferably inclined to side against the nurse, and "let the poor thing abide as she is." She must try with every art of tact and ingenuity, often for days, to effect some trifling object necessary to the wellbeing of the patient.

Lastly, it may be considered a truism to advise every nurse to choose a place suited to her. Why should a town-bred woman make her home where lonely lanes and open moors are a perpetual trial of nerve? Nor is there any need for the country girl to pen herself up in a manufacturing town, where her health and temper alike suffer. May I also, as a thick and thin North country woman, beg the Southerners to keep to their sunny South. Not to bring their tender chests and sensitive throats to our fierce blasts; where, too, the manners, habits, language, and peculiarities are not acceptable to them. Neither should the North country girl forsake her own bracing air, for the climate of the South is not suitable to her; and her blunt, out-spoken sentiments may very likely be misunderstood. A little thought would avoid all this, which is as bad for the work as for the nurse herself.

In reference to the mention of doctors, there is sometimes a possibility of bad feeling arising through the nurse outstepping her own boundary. Doctors do not like a nurse to take their work upon her; nor is it at all wise. She is not generally competent to give advice or suggest remedies, but to follow out lines laid down already. The poor are so anxious to get something gratis that they will prefer often to apply to the nurse; but they should not be encouraged, especially the thriftless ones, who belong to no "sick club." They ask nurse to "look in" as she passes. Perhaps a young nurse is quite taken in. She imagines nothing very particular to be the matter, and it does not occur to her that "a bit o' cold in his inside" is the usual definition of inflammation. So she gives some general advice as to keeping warm, and puts the matter off till her evening round. Then the doctor is sent for; but it is too late. The talk spreads, and the nurse must move on elsewhere, for the feeling both for and against her is too strong to allow of her doing any more good work in this place at least. Perhaps some may like to take this piece of experience—from real life—for what it is worth.

As to the remarks on our small kitchen operations, I have been told that they are too "philanthropically luxurious." I have therefore consulted other nurses, and I find their experience coincides with ours. For about 20s. a month—though some, I learn, have as much a week to spend—a great many little matters can be cooked if economy is studied. Lastly, as to the beds; one nurse tells me that she has had several patients at different times in her small cottage. They are generally children, and instead of waiting for a cottage hospital to rise magnificent on a lofty scale, she began work on the human element. She gave food from her table, and was allowed by her committee so much a week to defray the cost. "From small beginnings great endings." Perhaps if this simple plan were tried in other places, the hospital, instead of waiting year after year to be built, would grow up unconsciously in the midst of us, with its blessings of rest and peace to the sick and sorrowful.

### Nursing in Paris.

(By a Wandering Nurse.)

### FIRST IMPRESSIONS OF THE HOTEL DIEU.

Everyone who goes to Paris pays an early visit to the beautiful church of Notre Dame. Having seen the interior and all "the treasures" proudly exhibited by their custodian, most people pause to admire the fine exterior, afterwards allowing their attention to wander to the surroundings of the great mother church.

One of the nearest and most impressive buildings in this vicinity is the great hospital "Hotel Dieu," and this, of course, arrests the nurse-visitor at once. She soon turns her back on all other points of interest whilst she faces that entrance door.

Those who do not comprehend the fascination may, and do, smile at the infatuation of a nurse "on a holiday" wishing to penetrate within hospital walls. "What can Mary want to bother about hospitals for, when she is off duty?" remarks one of the family circle. "It's very silly of her!" promptly rejoins a younger critic.

A nurse, however, ventures to think differently. She knows that every new hospital through which she walks with observant eyes, forms a feature in her education. She has learnt to note how things should be done, and what arrangements can best facilitate skilled nursing. Hence she ought to be an intelligent as well as an accurate critic. If she finds a method to which she is unaccustomed, obtaining favour anywhere, she will not fail to enquire into the matter and to find out in what, if in any way, the novelty possesses advantages.

Frankly then, it must be owned that very few experienced nurses ever see the outside of a strange hospital without wishing to enter it.

Being, therefore, one fine September morning in the square in which both Notre Dame and Hotel Dieu are situated, I strolled instinctively from the former to the latter pile of buildings. It was not eleven o'clock, and a great quietness prevailed, so I boldly entered the stone passage and went up to a little group of three persons, one of whom was a trim-looking woman with a charming white frilled cap, the others were men evidently holding subordinate positions in the establishment.

I accosted one of the latter, but he turned towards the woman, evidently transferring my query to her, "Could I enter, and see something of her fine hospital," was a request received with little favour, so I proceeded to explain that I was a stranger, a sick nurse from England, &c. She replied with some asperity that it was the wrong time of day, and on my asking with much meekness what hour would be better, she vaguely suggested the afternoon perhaps, but warned me that my being allowed to see anything was problematic, although she granted that a "garde malade" had possibly a better chance than others.

My time in Paris was very short and therefore I concluded that instead of risking further disappointment I would endeavour to gain some professional advice and assistance. I had an introduction to a doctor who was said to be all-powerful and to possess infinite knowledge of institutions in general, but alas! he proved to be ill and away on a holiday. However, my courteous informant promptly furnished me with the names and addresses of two of the absentee's medical friends, and I hastened to the nearest of these.

How he managed to arrange it concerns no one but himself, but the result of our conversation was that soon after half-past eight next morning I found him quietly seated reading his daily paper under a tree in front of the great hospital. I followed him into the flagged passage (the wearer of the frilled cap was not there) and a grave-looking man received from my guide a rapid and apparently satisfactory explanation of my presence.

"Now that you are inside," he remarked to me in a low voice, "you may go where you will, you may witness all dressings, operations, &c., pass into any wards, penetrate amongst the outpatients, &c."

With satisfaction I looked around at the fine mass of buildings which enclosed the great courtyard, the long open galleries and stone bridges connecting the different departments. There were also, I found later, bright little gardens between some of the blocks, and assuredly all those patients who were able to leave their beds had every advantage of pleasant surroundings. Patients are admitted free, the doctor's judgment as to the urgency of the case being the only necessary qualification for entrance. The out-patients appeared to receive advice and medicine in rotation much as in our own waiting halls, and the men and the women's rooms are all separate. The east side of the hospital is devoted to male and the west to female patients.

My medical friend, to whom I shall always feel the deepest gratitude, spared time to give me a general view of the whole place before the arrival of his "chief." This was announced by the ringing of a bell at the entrance, and shortly afterwards an attendant threw open a ward door and announced the great man.

(*To be continued.*)

### Nursing in Ireland.

### COUNTY AND CITY OF CORK HOSPITAL FOR WOMEN AND CHILDREN.

This hospital contains 40 cots for children and 22 beds for women, and also a few private rooms which are very prettily furnished for the reception of patients paying an inclusive fee of two guineas a week for board, lodging, medical attendance, and skilled nursing. There are also semi-private wards containing only four beds, for which the patients are charged but one guinea each. For mental and massage cases there is, of course, an extra fee. The staff of trained nurses and probationers now number 30, and the accommodation for them has hitherto been very inadequate, but the matter is receiving the attention which it demands, and there is no doubt that in the hands of a committee so deeply interested in the welfare of their nurses and with a matron who has already done such great things for the hospital, the staff will soon have nothing further to wish for. A nurses' sitting-room is already near completion, and of the nurses' charming dining-room mention has been made in another column. Additional funds are needed, and contributions for the maintenance of the free cots and beds are urgently asked for.

### District Nursing.

(By an Association Superintendent.)

On reading an article in your last week's publication headed "District Nursing," I can only feel deep pity that the writer, "A District Nurse," has not had the great privilege which I have the honour to possess. I am one of Mrs. Dacre Craven's (Miss Florence Lees) oldest nurses, and as long as I live shall always be proud of having been trained in district nursing by her.

Miss Florence Lees never estimated a nurse's work by the number of cases or visits paid. Any extraordinarily high returns would only have gone to prove that the cases were but trifling ones, or else not properly nursed, according to her methods. I well remember her saying to me, "I do not judge any nurse's work by the number of visits or cases, but by the amount she does for each separate case." I have never forgotten these words, and have always worked on the same principle. After all, 2,965 visits in one year, when divided by 365, leave less than nine visits per day. I think we frequently paid as many visits, and often more.

Again, Miss Florence Lees may have ordered and expected us to do the "charing," but she always helped, taking the principal part of it herself; it was only done to show and instruct the friends of the patient how to do it, and how the room must be put in nursing order. Undoubtedly a nurse's influence with her patients and the neighbours is far greater if she actually does the cleaning herself, showing them that her profession and the training she has received does not make her less willing to do gladly what so many consider "menial," or, as the writer terms it, "manual work." I may be dull of comprehension, but I confess I do not understand either of these expressions when used in a discussion on the present subject. I cannot see that any work done from a right motive, and with the object of benefiting a sick person, can be called menial; and "manual work" I have understood to mean "any work which is performed by the hand." Where is the genius who can nurse without using her hands? The longer I remain in district nursing the more I am convinced that Miss Florence Lees was most practical and wise in her organisation of the work in London. It is all very well to be a good and clever nurse and carry out the doctor's orders skilfully, but surely something else is required to give the patient every chance of recovery. I mean the cleanliness and order of the room. An ordinary, uneducated woman (kind and good-natured as she may be) cannot be expected to know how to arrange the room, or why it should be done in one particular way, neither will she do it as quietly as the nurse herself. Once done by the nurse, the charwoman certainly may be allowed to keep the room clean, and the nurse need no longer distress herself about "spoiling her hands." I do not recollect that our hands suffered in the olden days any more than they do in the present, for we were encouraged to wear gloves while doing the rougher work, and to take great care of our hands at all times, in spite of the sweeping, dusting, and occasionally scrubbing, I frequently heard the patients remark upon the softness of "nurses' hands."

I admit that in the early days, when district nursing was so little known, we perhaps had more time to devote to each patient, and our superintendent's maxim being "thoroughness," even in the smallest things, we were encouraged by her to give plenty of time to each case, consequently our work was one of real pleasure, and not one of hurry and worry which, unhappily, is so often the order of to-day.

District nursing has become so universal, and the population of our large towns has so vastly increased, that more nurses are wanted every day, the supply does not nearly meet the demand, therefore a district nurse, especially a parish nurse, may not have so much time for "charing," if that is the orthodox term, and "if" her efficiency is rated by the number of visits she pays.

### Everybody's Opinion.

[Correspondence on all subjects is invited, but we cannot in any way be responsib'e for the opinions expressed by our correspondents. No communications can be entertained if the name and address of the correspondent is not given, or unless one side of the paper only be written on.]

### IS VIVISECTION JUSTIFIABLE?

"A Middle-aged Nurse" writes: In The Hospital for September 9th, in the review of a book by Ouida, this sentence occurs, "Vivisection must be justified or condemned on scientific grounds," &c. It seems to me that vivisection, like every other subject, must first be justified or condemned on moral grounds, and only when it is approved from the moral point of view may its desirability be further considered. I am far from wishing to discuss the subject of vivisection, but I must put in my protest against the assumption that it is merely a scientific question. Personally, I object to it on moral grounds.

### "NURSES AND DRESS."

"Another Professional Correspondent" writes: Will you allow me, through the columns of your paper, to protest against the very sweeping assertions made about nurses by the writer of the article on "Nurses and Dress"? I quite agree as to the unsuitability of nurses wearing trains either out or in doors, but as foolish people are to be found in all grades of workers there will always be some in the nursing ranks who cannot rise superior to the follies of their sex, and a walk through our streets will show that the train does not belong exclusively to nurses, as our correspondent would imply. As to outdoor uniform, although a few nurses may wear it for show, I know that many hundreds of earnest, hardworking women use it solely from motives of economy or convenience, certainly not with any desire to attract attention; and I think it is hard on the majority of nurses that they are to be dragged before the public and vilified because a few women are foolish enough to friz their hair and do various other foolish things that bring discredit on the whole profession. I wonder if it ever occurred to the writer of the article in question that possibly the nurses who go to the gallery of the theatre in uniform have no other attire to appear in? Nurses, as a rule, are not so highly paid that they can afford to provide worldly dresses which they have few opportunities of wearing save on their very brief holidays. The "Unprofessional Correspondent" raises an objection to the variety of cloaks, but why should ten thousand women wear one pattern? Is it more frivolous of a nurse to prefer nice, fresh strings than for other women to like birds or flowers in their hats? A nurse's bonnet is quite as much protection to its wearer as the headgear of any other lady, and surely it is no crime for the nurse to take womanly pleasure in a becoming uniform; but I quite agree with your correspondent that aprons and chatelaines are decidedly out of place in the street. There are still some nurses left whose heart is sufficiently in their work to make them indifferent to the frizziness of their hair, or the chance of catching a husband. It certainly is news to be told that the abolition of outdoor uniform will prevent flirting either in corridors or at the bedside! I think if matrons would refuse to keep useless women in their wards, and by example and precept set a high standard, we should hear less of nurses' shortcomings and more of their work; but so long as unsuitable women are taken on, simply because they pay, so long will complaints be heard. Many of those caricatures of nurses whom we meet in the streets are shams, and some have been dismissed as unsuitable; some have been a few weeks in lying-in hospitals, and then don our uniform and call themselves nurses! We hear a great deal about what nurses ought not to do, but what of the hundreds of Christian women who do good and brave work that the world never hears of? I know many such, and I cannot help thinking that the "Unprofessional Correspondent" lacks the sweet gift of charity when she writes that to pass unobserved in the street is "probably the last thing she wishes." It is a cruel and unjust statement. As to her discussion with the maidservant it is best treated with the contempt it deserves, though private nurses would do well to bear in mind that they go to help in the hour of need, not to be waited on as some seem to think. The objection to outdoor uniform as the possible means of conveying infection would apply equally to the dress of any other person, no conscientious nurse ever being found in the street in the dress she wears while in attendance on infectious cases, and there is no reason why she should be more likely to carry infection from outside than other members of the household where she is nursing.

### NURSING UNIFORMS.

Miss Mary Gardner writes: I should have thought that the last word had been said on the subject of nursing uniforms, but the letter of your "Unprofessional Correspondent" is so offensive in tone as to provoke a rejoinder from those who wear it. Kindly allow me space to answer the charges categorically. (1) "No lady other than a nurse would trail her skirts along a dirty pavement or a dusty floor." Nurses are not the only nor the greatest offenders in this respect. (2) "The noise which those sheaves of pencils, keys, scissors, &c., make jingling at the side." I believe that steel chatelaines are much less used than formerly, surgical cases having taken the place. But even where the chatelaine is worn I never found it so noisy an appendage. (3) "Outdoor uniform is apparently arranged not as a protection to the wearer but as an attraction." This I deny. The great majority of nursing uniforms are quiet in style and colour. Even if some are not in such good taste as others, yet the dress considered as a whole affords far less opportunity for display than ordinary costume. Noting especially the fashions of to-day, the glaring colours, often crude contrasts, and exaggerated styles, it is a relief to turn to the nurse's dress with its quiet, sober, uniform tints. (4) "White strings are added to the bonnet for show, and a long strip of gauze of various colours hangs down the back for effect, both arrangements being the most consummate shams and pretences." I thought that the white strings were merely intended to relieve the sombreness of black, dark navy, &c. I have never worn mine with any other idea. As for veils, they appear to find less favour with nurses themselves now, but if any like to retain them they may surely do so, as a becoming and graceful addition to the plain bonnet, and not altogether useless. If drawn round the throat and pinned they may form a serviceable protection on a cold day. (5) "Uniform is essentially a working costume, and it is therefore a gross piece of affectation when a nurse wears lt in public when the rest of the world is clad in the conventional evening garb." If a nurse wears uniform she generally keeps altogether to the same style of dress, and must consequently wear it wherever she goes. Her attendances at the theatre and opera are not frequent enough to make the expense of evening dresses for the purpose justifiable. We all know that the rank and file of nurses are poorly paid. The great advantages of outdoor uniform to a nurse are the saving of time and money thereby effected. To dress well and fashionably on a small income demands an amount of time, thought, and trouble, which nurses cannot well spare. Uniform, which is never out of fashion, but can be worn as long as the material will last, is a distinct saving. Nothing that can be said against it will outweigh the consideration of comfort, convenience, and economy. (6) "As a nurse, a woman is the observed of all observers." When outdoor uniforms were less common than now there might have been some truth in this assertion; but now that they are so generally adopted, the charge of conspicuousness breaks down. In half an hour's walk in London or any large city one meets twenty nurses, and the public has had time to become familiarised with their appearance. (7) "Another and very serious reason for the extinction of outdoo. uniforms is the danger to patients and public." I do not believe that the slightest danger exists. No nurses from infectious hospitals go out in the clothes they wear in the wards. The rules of these hospitals are very stringent. And the nurse from an ordinary medical or surgical ward will not carry infection. Moreover, the quarrel is with outdoor uniform, which is not worn in the wards. I am rather amused by the suggestion that some badge of the nursing profession might be worn under the cloak. If the cloak is to be retained, very little is left of nursing uniform with which to find fault. If hats were worn instead of bonnets, the effect would be, to nurses at least, somewhat incongruous, and hats might become as objectionable as even bonnets with veils. It is generally conceded that outdoor uniform is necessary for district nurses. I also think it desirable for private nurses attached to a hospital or nursing institution. Others, *i.e.*, hospital staff nurses and private nurses working on their own account, will decide for themselves. It they choose to wear uniform they have gcod reasons for doing so, and have as much right to please themselves in matters of dress as your unknown correspondent. Having myself worn uniform for more than seven years, I would not give it up on any account, for I should feel considerably perplexed if I were obliged now to study the fashions. Doubtless there are women in the nursing world who do not adorn their profession, whose motives and aims are unworthy. But of what body can this not be said? Our work is a noble one, and I believe that the majority of nurses will be found worthy of their calling. I hope we shall hear no more of such ungenerous and uncalled for attacks. We do not seek to interfere with others, with their dress, amusements, and occupations, and only ask liberty for ourselves in things not essential.

### PEDESTRIANS AND CYCLISTS.

"Pedestrian" writes: Whilst we are offering you our sympathy and condemning the particular cyclist whose careless conduct endangered your life and spoilt your hardly-earned holiday, we cannot restrain a few words on bicyclists in general from a pedestrian's point of view. So far, the Press has not given expression to this side of the question, having confined itself to the more personal one relating to the physical benefits conferred on wheelmen from this form of exercise; but the welfare of that portion of the public who yet are content to continue their course through life on the feet God gave them is equally important, and they have a right to speak also. For these speedy wheels, with all their vaunted immunity from harm to the possessor, have not the same right to a clear conscience regarding their effect on those unlucky persons who are not astride them. If one runs one's eye through the daily list of casualties caused through means of the so-called "safety" machine, one cannot fail to observe the manner in which their riders have usurped to their own particular selves the complete right to disregard any human obtrusion which may be in their paths; it has more than once, in the writer's own experience, seemed not beneath a cyclist's sense of honour to pass on at accelerated speed after knocking down a fellow creature, without proffering either assistance or apology. It is true that a warning bell is made compulsory by law, but how often do wheelmen wait until they are at your side before ringing it, when in a crowded thoroughfare the sudden chink-chink at your elbow paralizes your brain and makes retreat too late. It is high time that something should be done to insure the walking public against the ever-growing evil of careless bicycle riding; whilst a paltry fine of 10s. is an insufficient penalty to meet the seriousness of the case, wherein the victim to this latter-day wheelman's art finds himself broken limbed and incapacitated from work for many weary weeks.

### The Nurses' Looking Glass.

### ANNUAL HOLIDAYS—"TIMES OF REFRESHING."

"Times" or "seasons" of refreshing is an expression said to be taken from the custom of labourers who, in the heat of the day, repose themselves in cool shades, and surely we may use the words in reference to holiday-time. We might think of times of refreshing daily after the day's work is over, for there is a blessed limitation of labour, or we might consider occasional short holidays—days or half-days—as times of refreshing when daily work is awhile laid aside. It is not of blinks of refreshment in each day, or of short holiday times frequently occurring, that we are now specially thinking, but of the longer yearly holiday which falls to the lot of each member of an institution staff, and, of course, to many workers in other places. We will think now of our "Times of Refreshing," not in the day or half-day off duty, but in what is called the "annual," *i.e.*, the yearly holiday. That should be a season of refreshing at leisure—a holiday well earned by a year's spell at work. Our "annual" has, of course, its conditions; it must be applied for. There are many who can take their holiday when they like, but those in any kind of service cannot, whatever their station. The general cannot leave his military command; the admiral cannot leave his ship; the ambassador cannot leave his embassy, except on leave; no more can nurses and attendants; and this restriction is good. The holiday is not less enjoyed by those who go "on leave" than by those who can go when they choose. Again the "annual" has not only to be applied for, but to be arranged for, as to time. It must be convenient for others, as well as ourselves. Thus forethought is learnt in planning, and considerateness for others, in settling arrangements.

The duration of the yearly holiday is satisfactory; it is long enough to take in a fresh stock of health, spirits, and ideas, yet not over long so as to lose its zest. Persons after a holiday often say that they would have liked more, and if the holiday has been reasonably long, that is a good feeling to end it with. "Leave off with an appetite" is a good rule. In taking a holiday institution workers have not to provide for their work being done in their absence. It is taken up by work-fellows, as a matter of course; neither does the responsibility for the manner in which the work is done rest with the absentee. Contrast the case of members of the hardest worked of the professions. The medical man in private practice has very few, if any, "days off." If he wishes to take a holiday he may find it difficult to obtain a substitute, and have much anxiety about affairs during his absence.

When holiday time is arranged there is a pleasant interval of expectation, for hope is enjoyable as well as memory. Many a holiday would be more enjoyed if it were better planned, as regards companions, places to be visited, and the like. We may have seen in the holiday seasons little books on railway stalls or elsewhere with some such title as "Where shall we go to"? and different persons would answer the question, according to circumstances, in various ways. Some like to choose the popular watering places; at one 50,000 visitors were said to be present, which is quite natural after twelve months of monotonous routine work. Others prefer avoiding the more popular resorts and going further afield—to distant eastern or western watering places, or to some locality in Wales, Scotland, or Ireland, the Channel Islands, the Isle of Man, or some Continental town. In these more distant and less familiar expeditions, new information is acquired, and the mind is enlarged.

Short tours may be taken on foot, on cycle, on wheels, or otherwise along interesting routes, and with careful pre-arrangement these afford agreeable variety and fresh and pleasant experiences.

Again, there is the resort of one's own home—perhaps the home of childhood, of life-long remembrances. It is well to keep in close touch with that. The home may change so suddenly, and so soon. The old, aye, and the young, familiar faces may so soon vanish. Anyhow, visit the old folk as frequently as you can, perhaps dividing holiday time with them.

The well-spent holiday confers many benefits. Invigorating and enlivening the body by change of air and scenery; refreshing the mind by turning it away awhile from its ordinary monotonous current of thought, improving it by furnishing with knowledge and information, picked up amidst new scenes and associations, warming the heart by the renewal of home affections. Recreation is a very suggestive word, speaking of the renewal of body, mind, and spirits. We should also bestow benefits in holiday time. If we get good in our pleasant sojourn, here or there, we should also do good. We stay for our fortnight at a pleasant place without thinking of doing any good to the place which is benefiting us.

Of course, a visitor's presence itself may benefit the place of his (or her) temporary abode, its trade and industries. But this is not a disinterested benefaction. Again, a little considerateness and kind sympathy may be shown to those hard-worked lodging-house servants, whose attentions and civilities go so far towards making a holiday agreeable.

Or if the visit be to the Home, there should be a disposition not only to get all the affectionate attentions of those there, but also to cheer, to be of use, and to give pleasure to them. Young folk on returning home naturally like to be "made much of," but they should also endeavour to give comfort to those whom they visit.

The pleasure of a happy holiday does not end with it, for often amidst dull surroundings a fine view of cliff, river, or wood rises before the mind's eye, and also in recounting the past joys we can often share them with others.

### Notes and Queries.

SPECIAL NOTICE.

The contents of the Editor's Letter-box have now reached such unwieldy proportions that it has become necessary to establish a hard and fast rule regarding Answers to Correspondents. In future, all questions requiring replies will continue to be answered in this column without any fee. If an answer is required by letter, a fee of half-a-crown must be enclosed with the note containing the enquiry. We are always pleased to help our numerous correspondents to the fullest extent, and we can trust them to sympathise in the overwhelming amount of writing which makes the new rules a necessity.

### Queries.

(196) *Operations.*—Where can I get a book which would be helpful to nurses in preparing for operations?—*Nurse Maud.*

(197) *India.*—I want to know something about Lady Dufferin's nurses?—*J. P.*

(198) *Probationer.*—Where can I get particulars as to how to set about being trained?—*E. L. T.*

(199) *America.*—Information wanted as to how a nurse could obtain work in New York?—*San Francisco.*

(200) *A Born Nurse.*—I am very anxious to be a sick nurse when I get older, as all my friends say I am a "born nurse." At present I am in charge of a baby. What do you advise?—*J. B. M.*

### Answers.

(196) *Operations* (*Nurse Maud*).—Several good nursing books give this very fully. Do you know "Lectures to Nurses," by Eva Lückes, and "Handbook of Nursing," by Clara Weeks-Shaw?

(197) *India* (*J. P.*).—Write to Lady Dufferin and inquire.

(198) *Probationer* (*E. L. T,*).—Get "How to Become a Nurse," by Honnor Morten, published by the Scientific Press, 428, Strand, price 2s. 6d.

(199) *America* (*San Francisco*).—Many letters have appeared in The Hospital from time to time dealing with this subject. Read "England versus America" in February 18th, "American Experiences" in February 25th and March 11th, 1893; also various paragraphs respecting nurses in America which have appeared in The Hospital. You might advertise in the "Trained Nurse," published at 14, Vesey Street, New York.

(200) *A Born Nurse* (*J. B. M.*).—Do you know of the Norland Institute, Norland Square, London, where they undertake to train young girls as nurses in private houses? If you want to be a sick nurse, you had better write for "How to Become a Nurse." See answer to query 198.

### The Book World for Women and Nurses.

[We invite Correspondence, Criticism, Enquiries, and Notes on Books likely to interest Women and Nurses. Address, Editor, THE HOSPITAL (Nurses' Book World), 428, Strand, W.C.]

DUST AND LAURELS. By MARY L. PENDERED. (Griffith, Farran, and Co.)

A Study in Nineteenth Century Womanhood, so the authoress calls her book. The "study" centres itself upon two figures, representing totally different types of womanhood, with attributes and environments that are essentially modern. The dedication of her book, which is as follows, lends some clue to the purpose of the novelist: "To that hybrid complication, the woman of to-day, whose food is fruit of the tree of knowledge of good and evil, and whose drink is the intoxicating ether of freedom and independence." If Mrs. Pendered's object in writing this book was, at worst to disgust and at best to dissatisfy, we imagine she cannot fail to be pleased with the result of her work. "Dust and Laurels" is little else but a moral dissecting table. The first two pages of the volume provide the key-note. Witness the utterances of the heroine, Vera Grace, who says, in reply to her companion. "I mean this masterful power of touch, that unstrings all your resolutions and paralyses your intentions. The mere contact of a hand may send a darting fire up all your arteries, and seem to change your whole nature just when you feel strongest. Can you understand this?" Her companion replies, "I think so. The hand of some one you love?" "Oh, that is not what I mean at all! I mean something on a widely different plan; something you hate and loathe yourself for; something belonging to the masculinity of a man, that drives back the part you call your Soul, and makes you forget there is divinity in you. You are only conscious of sensation, and scarcely that. It is brutal," is the response that comes from Vera Grace. Now this young woman who thus unflinchingly and directly lays bare her not very edifying experiences is the peg upon which all the idiosyncracies of the modern woman, as Mrs. Pendered seems to know her, are hung. We cannot see that any of the other women in the tale are sufficiently strongly drawn to require remark. Miss Grace's career is not more edifying than her sentiments or sensations. She proves that a literary vocation is not sufficiently satisfying and absorbing for her, and after breaking one woman's heart by flirting —to speak mildly—with her husband, for whom she does not care, she marries the man she does not pretend to love, to save her from a worse man who exercises a strong fascination over her. All this may appear very modern in Mrs. Pendered's eyes. In our eyes neither that freedom at which she casts a stone nor these latter days are responsible. Even the authoress makes her heroine describe her early surroundings as bad; that, rather than the freedom which as a girl of the times she enjoyed, should account for the proclivities of Vera Grace. Modern freedom is rather responsible, we think, for the opera glasses with which Mrs. Pendered focuses our attention on a young woman whose bringing up, sentiments, and actions would be better left alone, but who existed as plentifully in earlier times as now, though she might not have been accidently recognised in society as Vera Grace appears to have been. We should regret to regard the more wholesome and reasonable education of modern

THE BOOK WORLD FOR WOMEN AND NURSES—*continued.*

women as lowering or futile as Mrs. Pendered depicts it. Rather we regard it as tending to produce a race of robuster beings, both physically and mentally, women who, standing side by side with their male companions, will make it possible that the best of all the talent the country can produce is utilised, not wasted. If the pendulum of womanly freedom swing a bit too far in the vigour of its youth, time and experience will intervene with controlling hands, and certainly we refuse to believe that freedom either in man or woman is another word for vice and error, as the teaching of "Dust and Laurels" would seem to indicate.

### SEPTEMBER REVIEWS.

Mr. A. S. Northcote gives a very cordial description in the Nineteenth Century of "American Life." He has studied it from many points of view, and has a word of good for almost every aspect of it, except perhaps the domestic servant. It is impossible not to feel in reading the "cook stories," with which American literature abounds, that a little want of humour is at the bottom of a good many of the grievances under which the American mistress labours. "The Southern story, so old, and told of so many people that it is a wonder the folklorists do not claim it as a sun myth, of the coloured lady who wanted to see 'de white woman ob de house 'bout de washin',' gives one an idea of the feeling among this class." Much of the home life is dominated entirely by the domestic difficulty, as an American woman's chief idea of happiness is to get away from her servants. In the watering places, "the centres of the community are the gigantic hotels, usually of wood, which rise at frequent intervals. Grouped all around them and often with little plank walks binding them together, and to the common centre-like cords, stand rows of tiny wooden cottages containing sleeping and dining accommodation for the family, but minus kitchen or servants' room. Meals are brought to their residents from the hotel, and from that centre are sent servants to attend to the wants of the cottagers. Thus the American housewife escapes for a few months from the tyranny of the servant girl, and can idly spend her days reclining in a rocking-chair on the hotel piazza, and discussing with her fellows the woes she has escaped and the wrath to which she must return." Meantime one wonders what becomes of the unhappy maids, and whether a little kindly deference (if that is their weak point) would be such a heavy price to pay for willing service and mutual attachment?

In "Glimpses Back, A Hundred Years Ago," a writer in Temple Bar quotes some curious extracts from the Annual Register relating to the doings of our ancestors in 1793. From the table of metropolitan mortality for the year it appears that the burials showed an excess over the christenings of 865, and that of the children who had died (the total deaths of all ages being 20,213) 8,703 were under five years of age, and that only 432 persons, male and female, reached the age of eighty in the twelve months. With regard to the proportion of infant deaths, since children were not counted till they had been christened, there would probably be many who died (born but unreckoned) before baptism. This suggests an appalling picture of infant mortality in London, and, indeed, elsewhere, only a hundred years ago.

The National Review maintains its usual high standard of interest. The short story by Mr. Frederick Greenwood can specially be recommended. It has all the charm and "sentiment" of a French study of life, full of delicate perceptions and artistically finished.

